# An automated workflow to screen alkene reductases using high-throughput thin layer chromatography

**DOI:** 10.1186/s13068-020-01821-w

**Published:** 2020-11-09

**Authors:** Brett M. Garabedian, Corey W. Meadows, Florence Mingardon, Joel M. Guenther, Tristan de Rond, Raya Abourjeily, Taek Soon Lee

**Affiliations:** 1 Joint BioEnergy Institute, 5885 Hollis Street, 4th floor, Emeryville, CA 94608 USA; 2 Biological Systems & Engineering Division, Lawrence Berkeley National Laboratory, Berkeley, CA 94720 USA; 3 Total Raffinage Chimie, 2 Pl. Jean Millier, 92400 Courbevoie, France; 4 Sandia National Laboratories, Livermore, CA USA; 5 Department of Chemistry, University of California, Berkeley, Berkeley, CA 94720 USA

**Keywords:** Thin layer chromatography (TLC), Geranylgeranyl reductase (GGR), Automation, High-throughput screening (HTS), Protein engineering, Isoprenoids

## Abstract

**Background:**

Synthetic biology efforts often require high-throughput screening tools for enzyme engineering campaigns. While innovations in chromatographic and mass spectrometry-based techniques provide relevant structural information associated with enzyme activity, these approaches can require cost-intensive instrumentation and technical expertise not broadly available. Moreover, complex workflows and analysis time can significantly impact throughput. To this end, we develop an automated, 96-well screening platform based on thin layer chromatography (TLC) and use it to monitor in vitro activity of a geranylgeranyl reductase isolated from *Sulfolobus acidocaldarius* (SaGGR)*.*

**Results:**

Unreduced SaGGR products are oxidized to their corresponding epoxide and applied to thin layer silica plates by acoustic printing. These derivatives are chromatographically separated based on the extent of epoxidation and are covalently ligated to a chromophore, allowing detection of enzyme variants with unique product distributions or enhanced reductase activity. Herein, we employ this workflow to examine farnesol reduction using a codon-saturation mutagenesis library at the Leu377 site of SaGGR. We show this TLC-based screen can distinguish between fourfold differences in enzyme activity for select mutants and validated those results by GC–MS.

**Conclusions:**

With appropriate quantitation methods, this workflow can be used to screen polyprenyl reductase activity and can be readily adapted to analyze broader catalyst libraries whose products are amenable to TLC analysis.

## Background

Alkene hydrogenation is an important process in petroleum refining, yet one that suffers pitfalls including catalyst poisoning, mass transport limitations, heat generation, and collective financial barriers associated with hydrogen, storage, and catalysts. For these reasons, performing alkene hydrogenation in vivo using engineered microbes has emerged as a potential alternative capable of overcoming these barriers [1]. Moreover, as synthetic biology tools and high-throughput screening (HTS) technologies become more affordable and accessible, microbes like *Escherichia coli* or *Saccharomyces cerevisiae* are increasingly utilized to biosynthesize commercially important molecules at industrial scales [2–7]. Hence, discovery of enzymes that can hydrogenate alkenes in microbes is of significant interest.

Enzymatic reduction of unactivated alkenes has been lightly studied, with limited past examples of biological isoprenoid hydrogenation [8–14]. Several enzymes classes that perform such chemistry include Old Yellow Enzyme, enone reductases, and fatty acid enoyl reductases [15–19]. One exemplary platform is a geranylgeranyl reductase isolated from *Sulfolobus acidocaldarius* (SaGGR). Its native function is believed to asymmetrically reduce all eight prenyl units within 2,3-di-*O*-geranylgeranylglyceryl phosphate, a precursor for archaeal lipid membrane biosynthesis [20]. SaGGR has been shown to reduce a variety of C_15_-C_20_ isoprenoid-based intermediates synthesized from either the mevalonate or DXP pathways [1], but SaGGR exhibits an optimum activity at ca. 55 °C, rendering it unsuitable for commercial biosynthesis in microbes such as *E. coli* or *S. cerevisiae* [21]. Hence, significant enzyme engineering is necessary to improve SaGGR’s properties for its use in engineered microbial strains to produce various saturated polyprenyl compounds.

Such engineering efforts logically require development of novel screening workflows to determine enzyme activities and product formation profiles associated with engineered GGR protein libraries. Current methodologies utilized for screening alkene reductase activity typically involve traditional, low-throughput methods including liquid chromatography (LC) or gas chromatography (GC) coupled to mass spectrometry. However, these low-throughput and analysis-intensive methodologies can significantly limit enzyme library screening throughput and lack considerable elements of automated handling and analysis. To this end, we turned to thin layer chromatography (TLC), an analytical technique routinely employed to rapidly separate and analyze small molecules from complex mixtures. Attractive features of TLC for SaGGR activity analysis include broad accessibility by laboratories without readily available mass spectrometry instrument time, instant visualization of product profiles from multiple samples in parallel, and robust readouts that eliminate the need for chromatogram integration. These advantages have led to development of TLC-based screens in a wide variety of applications, including catalytic antibodies [22], olefin metathesis [23], and olefin rearrangement [24].

**Fig. 1:**
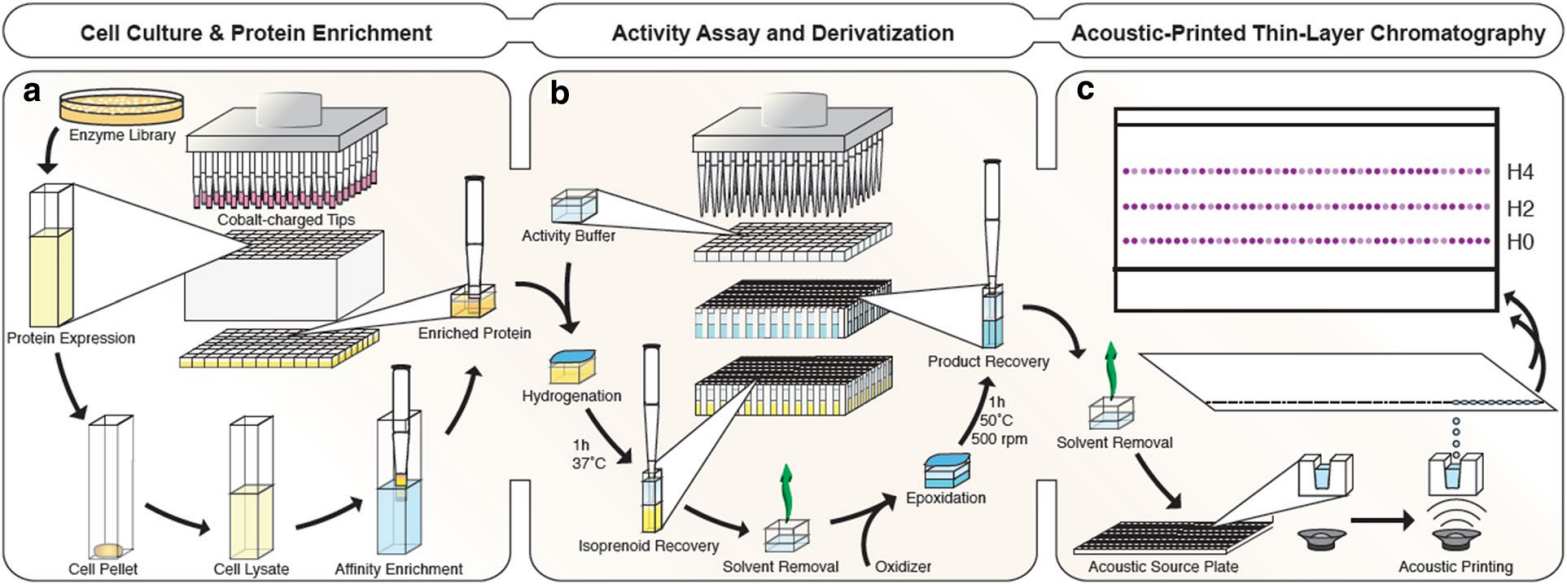
General scheme of the high-throughput assay for SaGGR. Each aspect was optimized with respect to **a** cell culture and protein expression, **b** assay formulation and product derivatization, and **c** mass transfer and product visualization

Herein, we develop a novel 96-well, TLC-based workflow capable of resolving isoprenoid mixtures with varying degrees of saturation. Prenyl groups that were not reduced by SaGGR were activated by epoxidation and coupled to the chromophore 4-(4-nitrobenzyl)pyridine (NBP) [25–27]. The epoxide functionality endows enzyme products with the polarity required for separation on normal-phase silica-TLC plates, while NBP selectively reacts with epoxides in a complex mixture, forming a distinctly purple product. Quantitative sample application is achieved by acoustic liquid transfer from 384-well plates directly onto TLC silica plates. Relative to traditional chromatographic methods, this workflow accelerates throughput and identification of SaGGR variants with improved reductase activities while adding the resolution needed to select for potential SaGGR variants with unique product profiles (Fig. 1).

We demonstrate the utility of this screening methodology to assess the relative reductase activities of SaGGR toward farnesol (FOH), a C_15_ isoprenoid harboring unactivated alkenes that is commonly used in fragrances, pesticides, and personal care products [28–31]. Partial or selective hydrogenation of farnesol by SaGGR has large potential to afford platform molecules for fine chemical, pharmaceutical, nutritional, and cosmetics markets [32–34]. However, the drastically low reductase activity of wild-type SaGGR prohibits its use in engineered microbes such as *E. coli* or *S. cerevisiae*. A variant of SaGGR at position Leu377 was previously shown to improve reductase activity towards geranylgeranyl pyrophosphate [21]. Hence, we employed the screening methodology developed herein to evaluate a codon-saturation mutagenesis library at this residue. Our workflow structurally resolves the fully unsaturated farnesol from enzymatically hydrogenated dihydrofarnesol and tetrahydrofarnesol products using normal-phase TLC. The product profiles of the library variants can semi-quantitatively distinguish among variants over a fourfold activity range, as cross-validated by GC–MS. While further development of software-based visualization tools can be employed to enhance the quantitative aspects of this screen, we present the basis of a plate-based workflow amenable to HTS automation that visualizes the challenging chemistry associated with enzymatic isoprenoid reduction in vitro.

## Results

### Automated SaGGR expression and purification

**Fig. 2:**
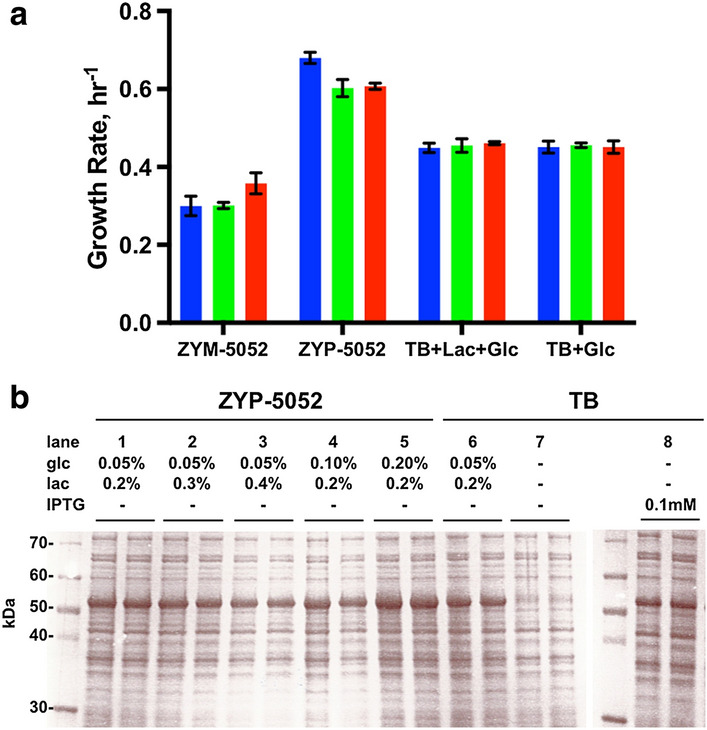
Optimization of 96-deep well cell culturing and protein expression conditions. **a** Various media were screened for cell growth by analyzing the log-phase growth rate as a function of glucose (glc) concentration (no glc, blue; 0.1% glc, green; 0.2% glc, red). **b** SDS-PAGE of SaGGR (MW = 53 kDa) present in soluble protein fractions cultured in ZYP-5052 supplemented with various lactose (lac) and glc levels. Lanes are run in biological duplicate with 0.05% glc and 0.2% lac (1), 0.05% glc and 0.3% lac (2), and 0.05% glc and 0.4% lac (3), 0.1% glc and 0.2% lac (4), 0.2% glc and 0.2% lac (5). These protein expression levels in ZYP-5052 were tested against TB medium induced with 0.05% glc and 0.2% lac (6), with no inducer (7) and induced with 0.1 mM IPTG (8)

A variety of cell culturing conditions were explored to optimize SaGGR expression in a 96-deep well format. We first compared the rich medium Terrific Broth (TB) against autoinduction media ZYP-5052 and ZYM-5052 under growth-phase conditions at 37 °C and for induction we supplemented all media with varying levels of glucose and lactose [35]. Cell cultures incubated in ZYP-5052 with 0.05% (v/v) glucose (ZYP-5052-glc) grew at the fastest rate among all growth media (Fig. 2a).

Protein expression levels were measured from 96-deep well cultures grown for 4 h at 37 °C up to OD_600_ of 0.5–0.7, followed by overnight expression at 37 °C. We examined expression conditions by supplementing ZYP-5052-glc with varying levels of glucose (glc) and lactose (lac). Most ZYP-5052-glc formulations expressed SaGGR at higher levels on the 1 mL scale than manually induced expression in TB media using 0.1 mM IPTG (Fig. 2b). SaGGR expression was generally unaffected regardless of glc/lac ratio, suggesting negligible *lacI* repression at lactose concentrations exceeding 0.2%. However, a higher glucose concentration, possibly delaying the induction, yielded higher levels of SaGGR expression. The ZYP-5052-glc 0.2%–lac 0.2% medium was thus selected.

**Fig. 3:**
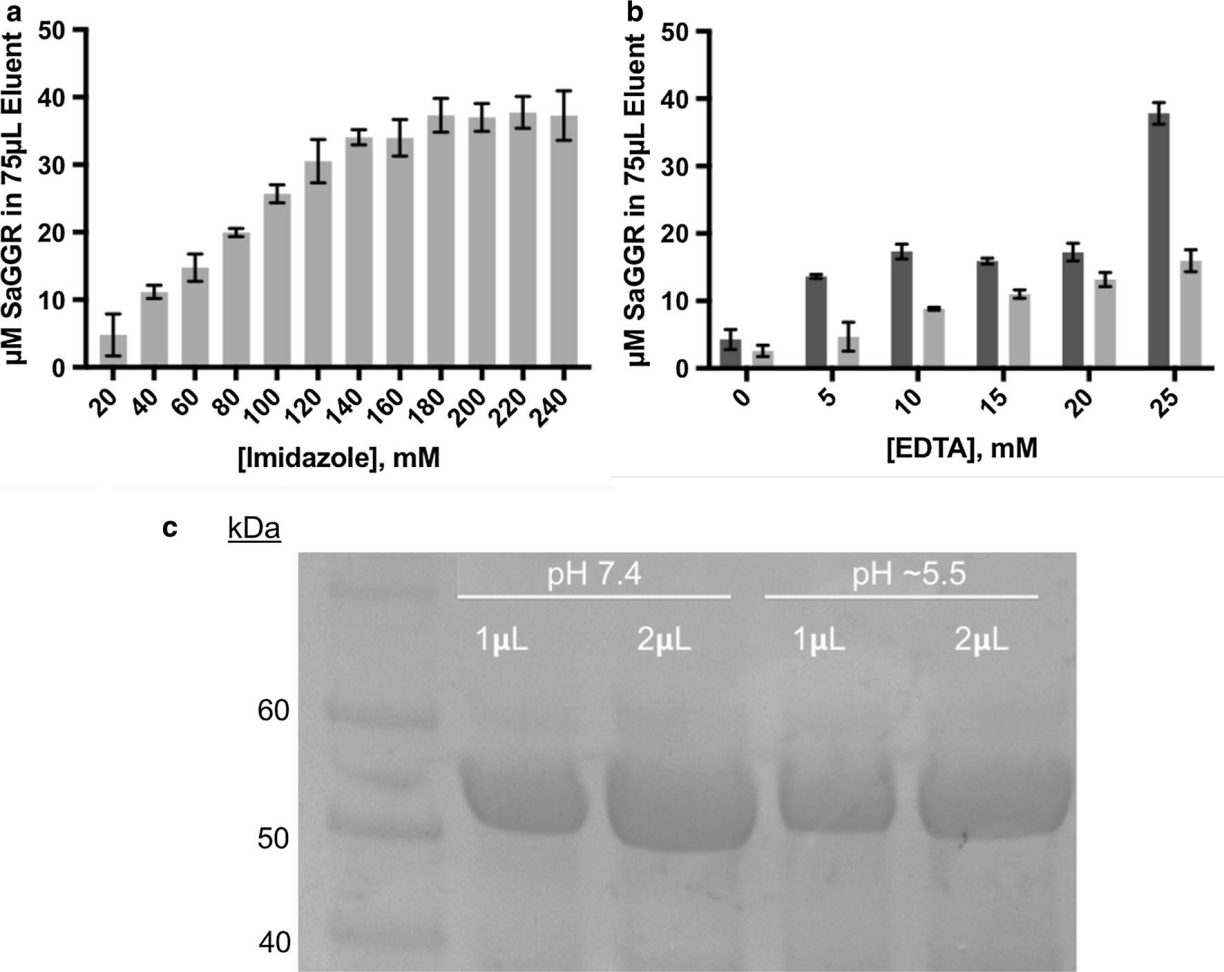
Optimization of the protein purification step. **a** Protein purification of GGR using IMAC tips for an automated 96-well format. Typical protein yields were 38 ± 3 µM GGR when using ≥ 180 mM imidazole. **b** Due to interference with downstream chemistry, purification was executed by stripping the co-functionalized tips with EDTA at pH 7.4 (dark grey) or pH 5.5 (light grey), where purifications utilizing EDTA at pH 7.4 is comparable to that of using imidazole. **c** SDS-PAGE showing the relative purity, stability, and yields of SaGGR upon elution (pH 7.4) and after buffer exchanging the eluent to the activity optimum (pH 5.5)

Several methods were explored to enrich SaGGR from cell lysates in a 96-well format. Based on its thermophilic origin, SaGGR remained soluble after heat treatment, unlike most of the *E. coli* proteome [21]. Incubation at 60 °C for 30 min at pH 7.4 enriched samples in SaGGR (Additional file 1: Figure S1). However, the recovered protein largely precipitated upon buffering to the enzyme pH optimum of 5.5. Instead, we opted for a 96-well resin-based enrichment using immobilized metal ion affinity chromatography (IMAC) tips (Fig. 3). Since imidazole-based elution interfered with downstream derivatization and isoprenoid recovery steps, SaGGR was eluted using ethylenediaminetetraacetic acid (EDTA) adjusted to pH 7.4. While enzyme elution was attempted at pH 5.5 to avoid further buffer exchange steps, purification yields were only comparable to that using 240 mM imidazole at pH 7.4. Under these conditions, SDS-PAGE analysis and protein concentration determination by Bradford assay revealed 75 µL of 38 ± 3 µM SaGGR could be obtained at > 90% purity and could be buffer exchanged to pH 5.5 without detectable precipitation (Fig. 3). Final concentrations of purified, pH-adjusted mutant proteins ranged from 0 to 75 µM.

### Enzyme activity assay and product recovery

Several formulation steps were optimized to activate purified SaGGR at its activity optimum at pH 5.5. Each 75-µL aliquot of purified GGR was buffered by incubation with a master mix containing the farnesol substrate and flavin adenine dinucleotide (FAD) cofactor dissolved in succinate buffer, followed by initiation of the enzyme reaction with sodium dithionite. Ultimately, this formulation (Methods) mimics in vitro assay conditions previously developed, but with increased substrate concentration [21].

**Fig. 4:**
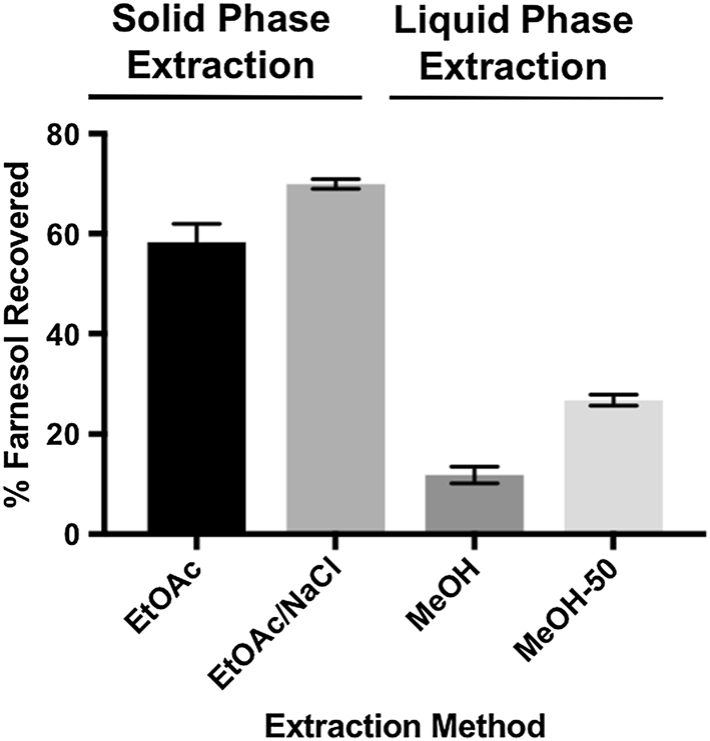
Comparison among various farnesol extraction and purification techniques following the enzyme activity assay. Liquid–liquid extraction techniques using ethyl acetate (EtOAc) or cold brine-saturated ethyl acetate (EtOAc/NaCl) were far superior for farnesol recovery in an automated 96-well format than solid-phase C_18_ methods utilizing methanol (MeOH) or 50% MeOH in EtOAc (MeOH-50)

Following enzymatic hydrogenation, recovery efficiencies of reduced isoprenoids from reaction mixtures were optimized using solid-phase or liquid–liquid extraction techniques. Optimal product recovery from 96-well C_18_ solid-phase extractions was achieved using a 50% (v:v) methanol:ethyl acetate (MeOH:EtOAc) elution mixture (Fig. 4). However, the low extraction efficiencies (< 30%) using a solid-phase approach were insufficient for subsequent derivatization and recovery steps. We then examined robotics-based liquid–liquid extractions using a 1:1 (v:v) addition of either EtOAc or EtOAc saturated in cold brine to the enzyme reaction mixtures, followed by organic phase recovery. Automated liquid–liquid extractions of the organic phase performed in 384-well plates was successfully achieved without disturbing precipitated mass accumulated at the organic–aqueous interface, subsequently increasing product recovery efficiencies to nearly 70% (Fig. 4). Following liquid–liquid extraction, recovered products were incubated in a chemical fume hood at room temperature until EtOAc solvent was fully evaporated.

### Reduced isoprenoid epoxidation

**Scheme 1:**
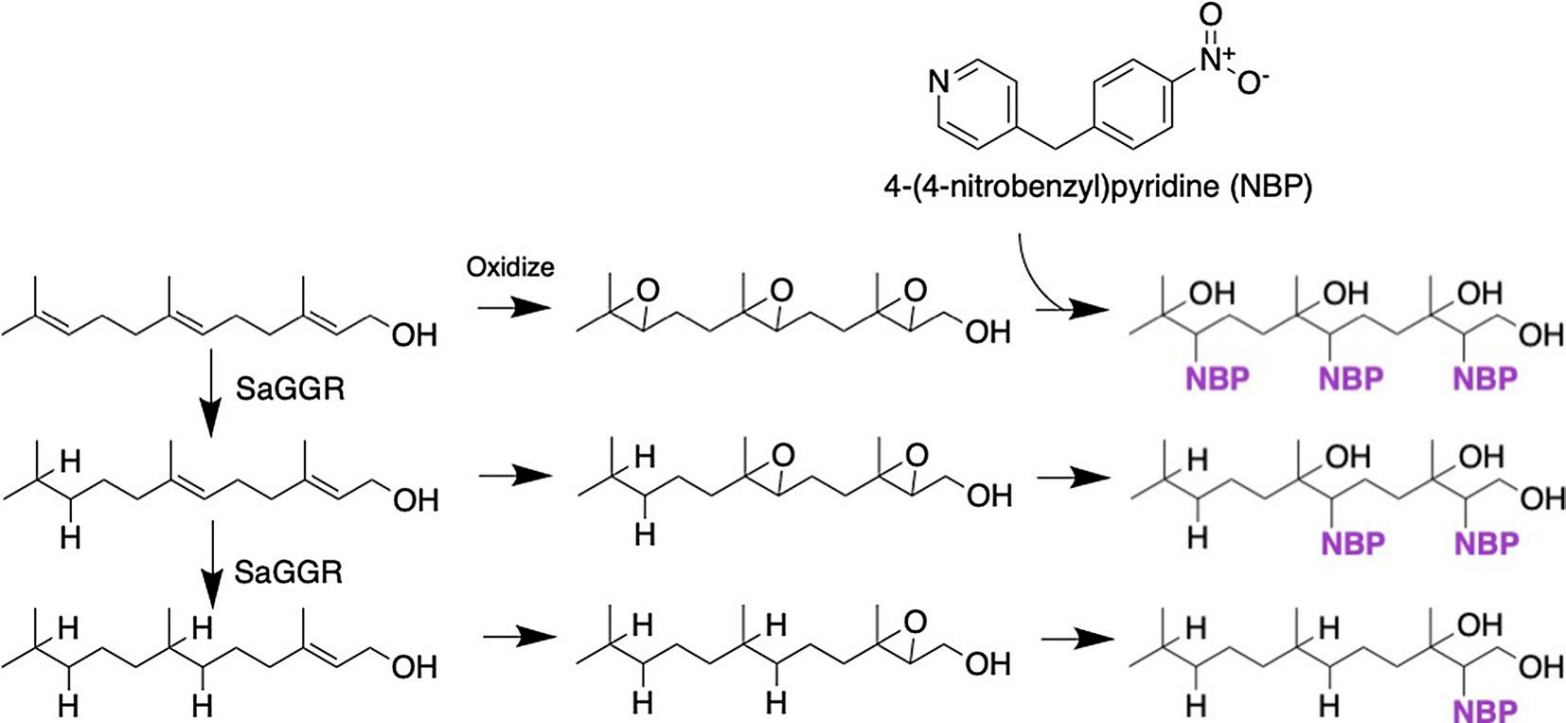
Overview of enzymatic farnesol reduction, isoprenoid epoxidation, and 4-(4-nitrobenzyl)pyridine (NBP) derivatized products utilized for screening

Isoprenoid derivatization was necessary to determine the extent of enzymatic farnesol reduction (Scheme 1). *(E,E)*-Farnesol was converted to mono-, di-, or tri-epoxyfarnesol in less than an hour when incubated with a 5% 2,2,2,-trifluoroacetophenone catalyst loading and six molar equivalents of H_2_O_2_ and acetonitrile (MeCN) at room temperature [26] (Additional file 1: Figure S2).

These epoxidation conditions, when applied in standard polypropylene plates in a 96-well format, were limited by apparent farnesol adsorption to the polypropylene wells (Additional file 1: Figure S3). We circumvented this issue by performing the reaction in glass-coated 96-well plates using vigorous stirring at an increased temperature of 50 °C. After incubation for 1 h, these reaction conditions yielded the expected mixture of epoxyfarnesol products that could be separated and visualized on the microliter scale (Additional file 1: Figure S3 and Table S1). The liquid–liquid extraction procedure using equal volume of EtOAc established for product recovery was sufficient for recovery of epoxyfarnesols from aqueous solution. EtOAc was again fully evaporated by incubation at room temperature and the epoxyfarnesol products were reconstituted in a transfer buffer amenable to mass transfer by acoustic droplet ejection.

### Acoustic droplet ejection and thin layer chromatography

**Fig. 5:**
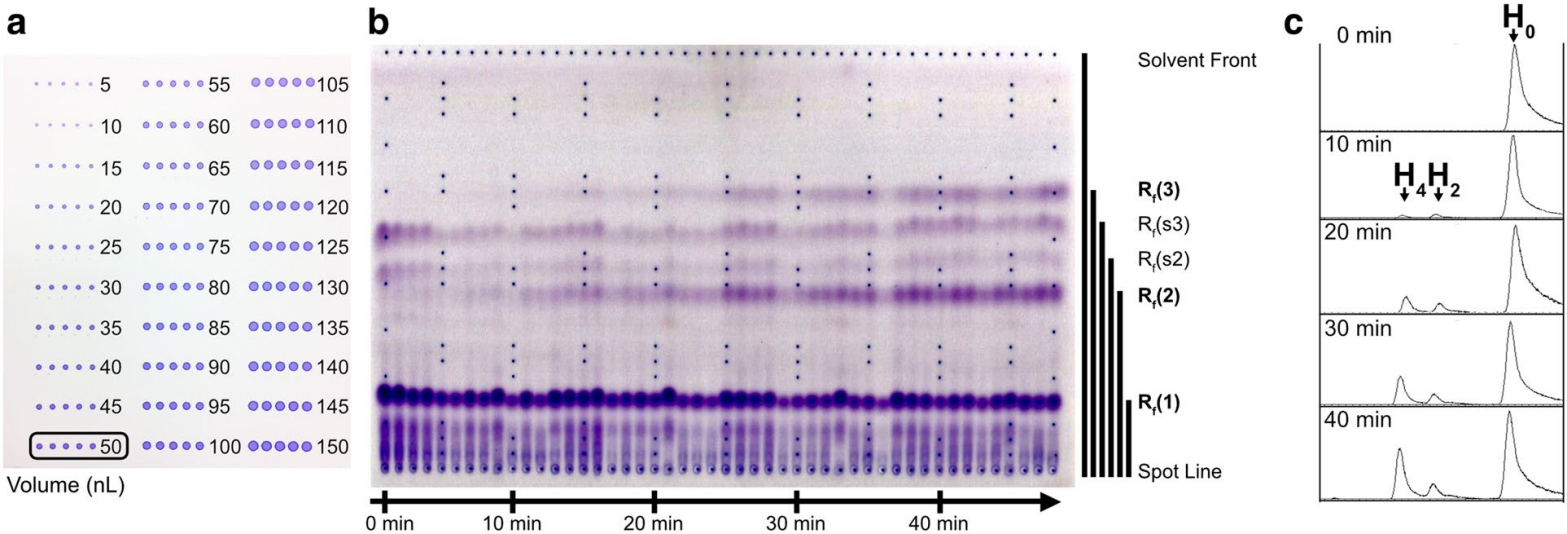
Optimization of acoustic droplet ejection and application of acoustic printing for the time-course assay. **a** Nanoliters of coomassie stain transferred by acoustic droplet ejection to a silica-TLC plate. The optimal volume at 50 nL, circled, was determined as the best volume for a simultaneous 48-well TLC analysis. **b** A time-course assay of 30 µM WT SaGGR incubated with 2 mM farnesol quenched with epoxidation reagents every minute and spotted in a separate lane. As the assay evolves to its 48-min endpoint, the bands corresponding to the epoxide derivatives of H_0_-, H_2_- and H_4_-FOH are indicated by *R*_*f*_ (1), *R*_*f*_ (2) and *R*_*f*_ (3), respectively, and increase in intensity while side-product bands *R*_*f*_ (s2) and *R*_*f*_ (s3) remain relatively constant with time. Quantitative *R*_*f*_ values for all bands are listed in Additional file 1: Table S1. **c** Gas chromatograms derived from the time-course described in **b** depict farnesol (H_0_), di- (H_2_) and tetrahydrofarnesols (H_4_)

**Fig. 6:**
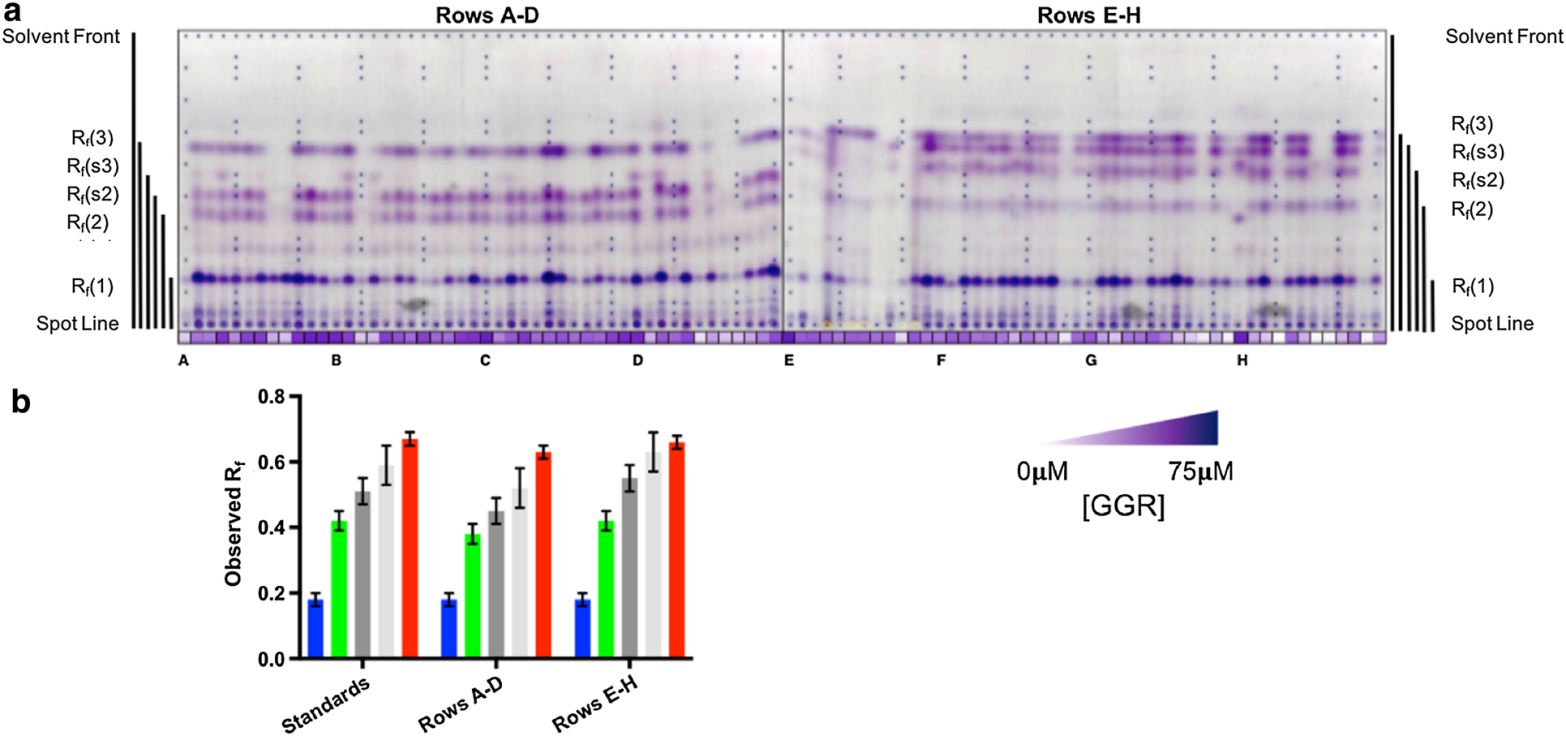
Application of the screen to a codon-saturation mutagenesis library, L377X. **a** TLC plate for product analysis. The first 48 lanes on the left represent activities in mutants contained in rows A–D of a 96-well plate format; the other 48 lanes on the right represent activities in mutants contained in rows E–H. Protein concentration measured in each well via Bradford assay is denoted under each TLC lane. Following chromatographic separation, NBP-treated plates were heated in an oven at 150 °C for 10 min and developed with triethylamine. **b** Comparison of *R*_*f*_ values observed in the time-course assay (Fig. 5) denoted as standards with observed *R*_*f*_ values in rows A–D and E–H within the mutant library. While the observed side products *R*_*f*_ (s2) and *R*_*f*_ (s3) migrate slightly differently among plates (dark grey and light grey, respectively), the respective products corresponding to FOH (blue), H_2_-FOH (green), and H_4_-FOH (red) at *R*_*f*_ (1), *R*_*f*_ (2), and *R*_*f*_ (3) migrate within error among all TLC plates

To directly compare product profiles between enzyme variants, we developed a novel liquid transfer method using acoustic droplet ejection technology to apply epoxidized reaction products onto thin layer silica. Epoxyfarnesols were reconstituted in MeCN:H_2_O (2.5:3), a ratio optimized to sufficiently dissolve products while maintaining a viscosity amenable to liquid transfer. After loading products into an acoustic source plate, samples were centrifuged to homogenize interactions between the source plate and the transferring liquid and facilitate optimal accuracy of sample ejection volumes. Repeated application onto silica enhanced robustness of product visualization, but had to be balanced with droplet over-spotting, which led to increased band spreading and loss of resolution between lanes. To reduce over-spotting effects, ejection volumes of 48 wells were minimized to 50 nL and transferred iteratively until all reaction products were applied (Fig. 5 and Additional file 1: Figures S3, S5). Following product application, a second acoustic droplet ejection was performed to create a Coomassie-stained grid that assisted in visualization of running lanes and calculation of relative retention factor (*R*_*f*_) values (Additional file 1: Figure S4). The acoustic droplet ejection process was repeated onto a second TLC plate for the remaining 48 wells of the 96-well plate for full plate analysis. The mobile phase was optimized to 2% MeOH (v/v) in EtOAc:hexanes (1:1.25), and neither mobile phase nor applied sample showed appreciable interaction with the Coomassie stain. Following product resolution by TLC, plates were treated with the pro-chromogenic NBP. Preliminary attempts to ligate epoxides with NBP at 100 °C afforded product bands with faint color intensity (Additional file 1: Figure S5). Accordingly, plates were treated with NBP and heated to 150 °C for 10 min and treated with triethylamine to markedly improved color intensity (Figs. 5 and 6), signifying the formation of the epoxide–NBP adduct.

*R*_*f*_ values corresponding to epoxyfarnesols were determined by a time-course assay. SaGGR was incubated with farnesol and cofactors, and samples were extracted at 1-min intervals for 48 min and quenched by incubation with epoxidation reagents (Fig. 5). Five products (**1**, **2**, **s2**, **s3** and **3**) were observed during the time-course, with two putative product bands systematically increasing in intensity with time (Fig. 5b). The H_0_-FOH derivative, tri-epoxyfarnesol (**1**), migrates the least with a *R*_*f*_ (**1**) = 0.17 ± 0.02. Its strong intensity remains, suggesting the reaction is not substrate-limited, also seen by GC analysis (Fig. 5c). The epoxide derivative of H_2_-FOH, dihydrofarnesol diepoxide (**2**), appears after approximately 10 min, migrating to a *R*_*f*_ (**2**) = 0.43 ± 0.03 and can be seen by either TLC or GC. After 25 min, the tetrahydrofarnesol (H_4_-FOH) product is formed, and its derivative, tetrahydrofarnesol monoepoxide (**3**), migrated to a *R*_*f*_ (**3**) = 0.67 ± 0.04. Despite previously establishing conditions for the synthesis of product **1** at the microliter scale, uncharacterized side products **s2** and **s3** routinely appeared at higher *R*_*f*_ values. Each of the two side products are proposed to arise from off-target farnesol oxidation, producing side products with measured *R*_*f*_ values at *R*_*f*_ (**s2**) = 0.51 ± 0.04 and *R*_*f*_ (**s3**) = 0.60 ± 0.04. The reaction time-course, which was sampled prior to epoxidation and analyzed in parallel by GC–MS, indicates time-dependent increases in products with mass spectra corresponding to di- (H_2_-FOH) and tetrahydrofarnesols (H_4_-FOH) (Fig. 5c). While our previous GGR characterization study indicated mass spectral peaks corresponding to low levels of oxidized polyprenyl alcohols and polyprenyl phosphates [1], no side products were observed in this study by GC–MS, nor were they detected in commercial farnesol. Together these data suggest off-target oxidation stemmed primarily from epoxidation conditions performed in 96-well plate format. In line with previous activity measurements, these oxidized side products appear at the zero timepoint in the kinetic analysis and remain relatively constant throughout the time-course, suggesting that they form independently of products **2** and **3** and SaGGR activity evaluation would not be affected by them. Another consideration is the potential for acid-catalyzed epoxide ring opening to yield mixed glycol products, and especially true under the acidic conditions on TLC silica gels. In this case it should be possible to visualize mixed glycol epoxides using the epoxide-specific stain, NBP. Epoxyfarnesol retention factors remained constant and product spots were homogeneous, suggesting this pathway does not occur to an appreciable extent (Additional file 1: Figures S2, S3). Finally, preliminary experiments indicated epoxidation to proceed under the assay conditions in 1 h, though it is possible these side products represent incomplete epoxidation of unreduced farnesol to mono- and diexpoxy farnesol species. Nevertheless, the interferences by these side products are well-resolved from the derivatized, hydrogenated farnesol products of interest.

### Library screening for SaGGR isoprenoid reduction

Application of the complete workflow to a codon-saturation mutagenesis library was executed in a 96-well format. Demonstration of this workflow targeted a leucine at position 377 (L377X) in SaGGR (Fig. 6), a site where various mutants demonstrated increased reductase activity toward GGPP [21]. Ninety *E. coli* clones producing a L377X variant of SaGGR were randomly selected and inoculated in a 96-deep well plate along with 3 negative controls (*E. coli* strain containing a plasmid without a GGR gene) positioned in the A1, D5, and H11 wells, and 3 positive controls (*E. coli* strain expressing the WT SaGGR gene) in A2, D6, and H12.

As expected, negative controls did not reveal any visualized products, and WT wells revealed five product bands with *R*_*f*_s in line with those calculated during the time-course assay (Fig. 6). Collectively, the screen revealed six product bands in the TLC profiles, including an additional faint side-product (*R*_*f*_  = 0.26 ± 0.02) observed in many of the mutants contained in A–D wells (Fig. 6 and Additional file 1: Table S1). Notably, this side-product could be the diepoxy adduct resulting from the regioisomer of H_2_-FOH in which the middle prenyl group is reduced instead of the terminal one as proposed in the previous GGR characterization study [1]. Retention factor values for non-enzymatic side products (*R*_*f*_ (**s2**) = 0.51 ± 0.04 and *R*_*f*_ (**s3**) = 0.60 ± 0.04) were shifted slightly downward in the A-D plate (*R*_*f*_ (**s2**) = 0.46 ± 0.03 and *R*_*f*_ (**s3**) = 0.52 ± 0.04). This difference was attributed to differences in headspace saturation within the TLC chamber after running the E–H plate, as similar shifts were not observed in preliminary attempts to screen the L377X library using this workflow (Additional file 1: Figure S5).

Nonetheless, the FOH, H_2_-FOH, and H_4_-FOH epoxy adducts ran at reproducible *R*_*f*_s within error of one another in all cases (Fig. 6 and Additional file 1: Table S1). Hence, we assessed activity based on these product band intensities relative to the measured protein concentration. While the in vitro protein expression levels in each assay ranged from 0–75 µM, the relative product band intensities among proteins with comparable expression varied considerably (Fig. 6). Some mutants exhibiting poor protein expression in wells including A8-A9, B3, D6, G10, and H7-H8 revealed no intensity within the product bands. Other wells with robust protein expression such as C11 and E1 also revealed little product formation relative to other mutants. On the other hand, some mutants (C7, C10, and H9) produce robust product intensities while having low levels of protein expression, suggesting improved reductase activity. Taken together, these observations suggest the workflow does not suffer from biases associated with location within the 96-well plate.

**Fig. 7:**
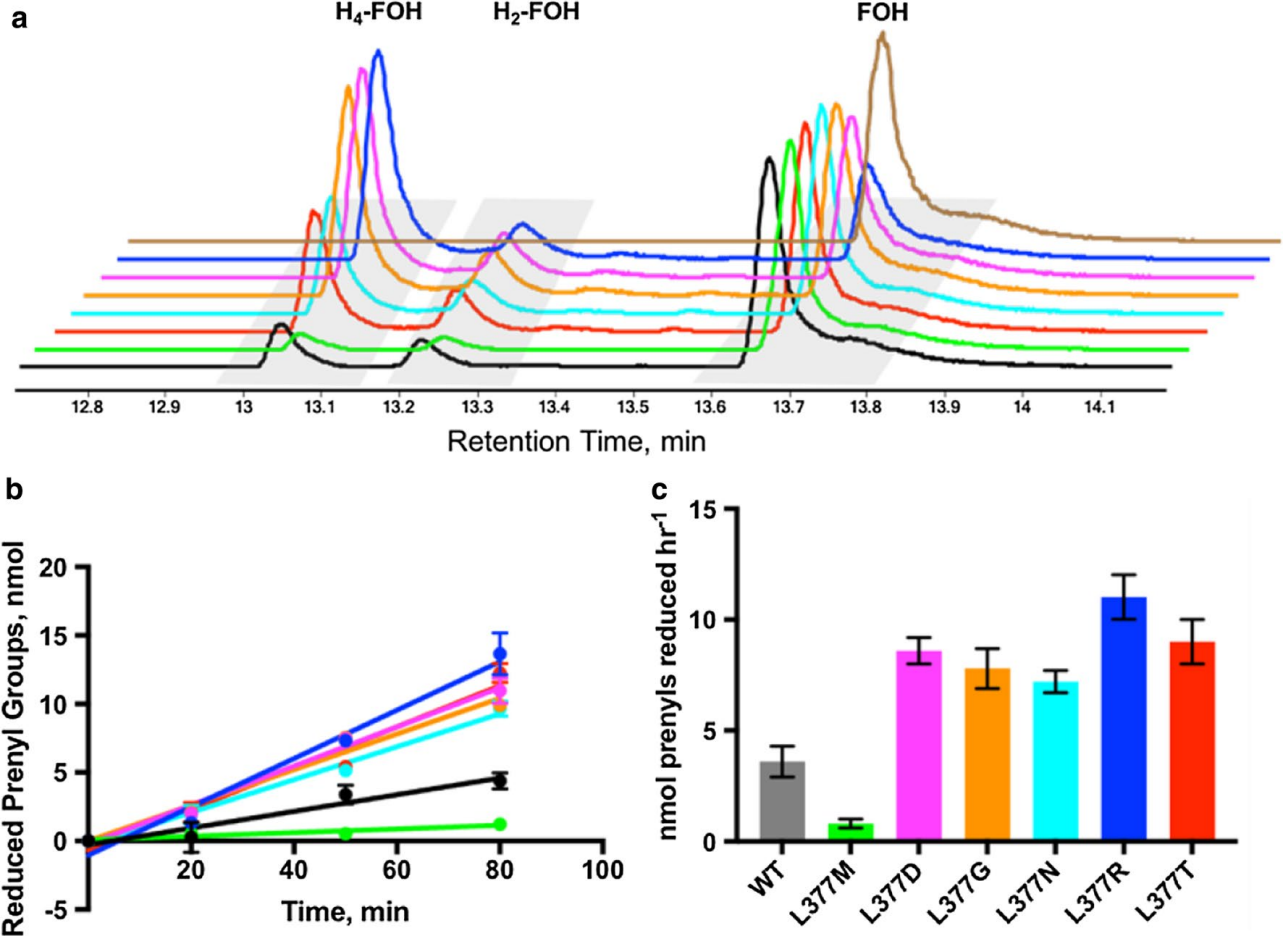
Comparison of select mutants within the L377X library. **a** Normalized GC–MS traces of products isolated after 80 min of incubation time with 30 µM WT SaGGR (grey), L377M (green), L377D (magenta), L377G (orange), L377N (teal), L377R (blue), or L377T (red) with 500 µM farnesol at pH 5.5 and 37 °C. **b** Time-course comparing product formation among all mutants at 0, 20, 50 and 80 min. **c** Relative turnover numbers for all mutants. The color schemes used in the bottom panels are consistent with that used in the top panel

Various mutants exhibited a range of activities within the TLC screen. Hence, we proceeded to investigate how the qualitatively visual differences among band intensities observed in the TLC screen correlated to enzyme activities measured using traditional GC–MS techniques. We ran the assay under previously established in vitro assay conditions to more accurately quantify product formation to correlate its performance with the visual features observed within the screen’s viability (Table 1). We selected the B2, C7, C10, E1, E9, G11, and H7 variants based on several factors, including product band intensities, protein expression levels, and diversity of location within the 96-well plate. The genes encoding the variants were sequenced; B2 (L377T), C7 (L377N), C10 (L377G), E1 (L377M), E9 (L377D), G11 (L377R) and H7 which encoded a stop codon at L377. In accordance with the product profiles observed within the TLC screen, standard assay conditions (30 µM enzyme, pH 5.5) revealed a range of reductase activities toward farnesol (Fig. 7 and Table 1). All sense-coding mutants except for the E1 (L377M) mutant exhibited a modest 2–3 × increase in prenyl reductase activity relative to wild type (3.6 ± 0.7 nmol prenyl groups reduced h^−1^), reflective of the increased product band intensities within these TLC lanes (Fig. 7 and Table 1). Except for C10 (L377G), residues exhibiting increased activities were mutated to either a polar (Thr or Asn) or charged (Asp or Arg) side chain. Surprisingly, the most conservative mutation to a hydrophobic methionine side chain within the E1 well position exhibited a ca. sixfold decrease in activity. While L377M exhibits robust protein expression under the deep-well culturing protocol, only the substrate band is visualized within the TLC plate. Hence, these activities measured by traditional assay methods have validated the utility of a semi-quantitative protein screen for prenyl reductase activity, as increased product band intensity appears to correlate with increased enzyme activity.

**Table 1:** Overview of selected mutants from the L377X library

Well	Codon Substitution	Amino Acid Substitution	Specific Activity, (nmol Prenyl Groups Reduced h^−1^)
A2	CTG	WT	3.6 ± 0.7
B2	ACG	L377T	9 ± 1
C7	AAT	L377N	7.2 ± 0.5
C10	GGG	L377G	7.8 ± 0.9
E1	ATG	L377M	0.8 ± 0.2
E9	GAT	L377D	8.6 ± 0.6
G11	CGT	L377R	11 ± 1

## Discussion

The inherent chemistry associated with detecting alkene hydrogenation vastly limits the techniques suitable for automated workflows. Even though synthetic approaches have shown significant progress in this regard, there are also some limitations [36–38]. For example, while the reduction chemistry of libraries containing heterogeneous catalysts has been successfully demonstrated on a 96-well scale in parallel, product analysis remains a significant bottleneck. Either accelerated chromatographic methods are needed for product detection, or chemometric approaches are needed to assign signatures within heterogeneous samples using combinations of techniques such as infrared thermography, X-ray spectroscopy, or fluorescence to determine product formation [39].

In biological systems, enzymatic alkene reduction has been studied in HTS fashion on vinyl groups adjacent to the electron withdrawing group (i.e., $\alpha ,\beta$-unsaturated carbonyls) using Old Yellow Enzyme [40]. However, this system indirectly measured NAD(P)H consumption via a well-known glucose oxidase-based colorimetric method using activated alkenes as substrates. SaGGR has been proposed to receive reducing equivalents from ferredoxin and cannot perform reduction chemistry using NAD(P)H [12]. Hence, the only suitable means to detect enzymatic olefin reduction by SaGGR is to monitor the presence of its substrate and products. HTS methods have successfully demonstrated alkene reduction to chiral compounds in the case of styrene oxidase mutant libraries using NBP as a colorimetric derivatization agent [25]. However, NBP derivatives quickly decompose in solution, resulting in a colorless product after 20 min under typical screening conditions [41, 42]. Moreover, reduced farnesol products formed upon incubation with SaGGR contain fewer sites for NBP derivatization than farnesol, further emphasizing the increased adduct stability needed for product visualization.

Because of the inherently low specific activity of SaGGR, several process-oriented steps required significant optimization for execution on a 96-well scale. Several micrograms of SaGGR enzyme per well were needed to produce a detectable amount of reduced farnesol products. To achieve this conversion, high concentrations of enzyme were required to maintain low reaction volumes. The large amounts of enzyme loadings were strongly dependent on medium selection, culturing conditions, and protein expression and purification parameters during the automated processes (Figs. 1–3). Following enzymatic reduction, product lability rendered aqueous absorption measurements ineffective, motivating our TLC-based approach that allows us to monitor both substrate and products in a complex mixture. In order to achieve a reliable endpoint readout, conditions were optimized for maximal product recovery and derivatization yields, along with efficient mass transfer (i.e., acoustic droplet ejection) to successfully apply the minimum amount of product needed (ca. 1 nmol) for robust TLC visualization and resolution (Figs. 4–6).

The modularity of this biochemical screen allows for straightforward modifications to enzyme reaction parameters (e.g., pH, reaction incubation time, temperature, etc.) as improvements are achieved. In contrast to indirect methods typically employed to evaluate enzyme activity (e.g., enzyme-coupled assays or engineered pathways utilizing optical biosensors), this screen exhibits the resolution to separate multiple products (Figs. 5 and 6). Unlike solution-phase colorimetric assays, this TLC-based screen can identify mutants that can selectively form each of the FOH, H_2_-FOH, and H_4_-FOH products. Moreover, the excellent resolution between lanes allows an entire 96-well plate to be visualized on two 8 × 12 cm plates for simple visual inspection of enzyme library hits. In principle, this screen can be adapted for quantitative applications with the appropriate visualization software packages to quantify the amounts of each product present within each mutant well. In addition, this workflow can be adapted to evaluate broader catalyst libraries whose products are amenable to TLC analysis. Nonetheless, this workflow highlights distinct qualitative differences in reactivity and can provide increased insight among various GGR mutant libraries.

To this end, we successfully demonstrated the utility of this novel TLC-based screen on a codon-saturation mutagenesis library on the Leu377 of the SaGGR protein (Fig. 6 and Additional file 1: Table S1). The limited L377X library revealed a wide range of activities, with several mutants revealing intensity variations in both H_2_-FOH and H_4_-FOH bands relative to WT. Moreover, mutants with varying activities could be distinguished by inspection, without the need for traditional MS-based methods. L377M and WT exhibited significantly lower product band intensities relative to L377G, L377D, L377R, L377T, and L377N, and were unaffected by biases based on 96-well plate position. Because the range of mutant activities visualized by TLC were reflective of activities quantified using MS-based methods, this screen showcases the ability to quickly identify variants with improved prenyl reductase activity (Fig. 7 and Table 1). To our knowledge, this is the first approach that enables increased throughput to detect enzymatic reduction of unactivated hydrocarbons. With appropriate synchronization, we estimate an upward throughput of five to ten 96-well plates per day, affording far greater throughput than methods currently available to screen prenyl group reduction semi-quantitatively.

**Fig. 8:**
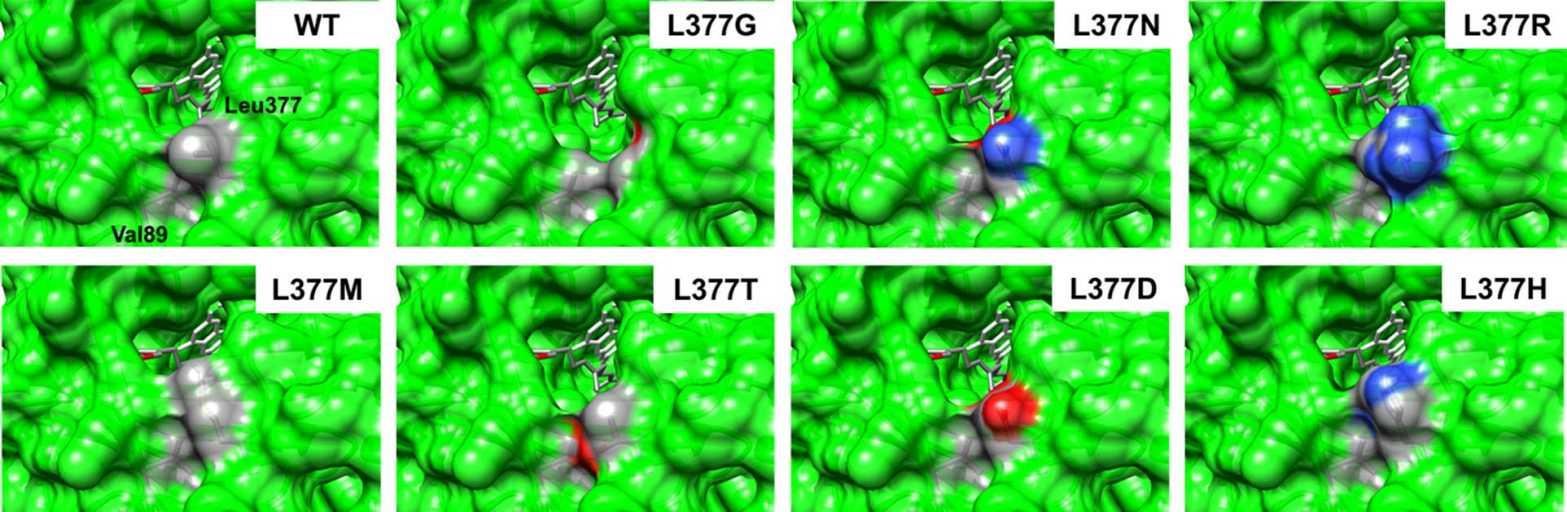
View of the active site from the entry point of the substrate-binding tunnel. Leu377 from the substrate-binding domain and Val89 from the catalytic domain in WT SaGGR form van der Waals interactions between methylene groups (grey) that comprise the tunnel’s native contacts. This tunnel appears to be occluded in the L377M mutant, which is the only mutant shown to decrease reductase activity toward farnesol. In the remaining mutants enhancing reductase activity, they either expand the substrate-binding tunnel (L377G or L377T) or provide charges that could enhance protein–solvent interactions via hydrogen-bonding oxygens (red) or nitrogens (blue) (L377N, L377R, L377D, L377H)

In silico models of the L377X mutants tested reveal very little direct enzyme–substrate interaction. Moreover, the only potential contact Leu377 makes with any other residues involves weak, solvent-exposed van der Waals interactions with Val89 (Fig. 8). This is one of several surface interactions between the catalytic and substrate-binding domains that form the substrate-binding cavity [12]. L377M, the only residue that exhibited decreased activity relative to WT, appears to partially seal this proposed cavity by its increased size and enhanced interaction with Val89. Crystal structures containing the notably larger GGPP substrate reveal enhanced interactions with the pyrophosphate moiety upon mutation of Leu377 to histidine, leading to enhanced turnover [21].

For enhanced turnover on farnesol, however, the remainder of the mutants appear to enhance reductase activity by either eliminating hydrophobic bulk (L377G), or by adding residues with polar or charged side chains (L377N, L377R, L377D, L377T). Models using the highest probability rotamers without van der Waals clashes show that all charged and polar moieties are oriented toward the solvent interface except for L377T. However, the hydroxyl side chain is within hydrogen-bonding distance of the backbone carbonyl of Glu381 (Fig. 8).

The contribution of increased polarity at residue 377 to SaGGR reductase activity is unclear. However, crystallography data on a related archaeal GGR isolated from *Thermoplasma acidophilum* have shown that residues encompassing the Val89/Leu377 interaction belong to the most flexible region of the enzyme [13]. This flexibility is proposed to enhance substrate binding and product release for the native substrate, digeranylgeranylglyceryl phosphate (DGGGP). While further biophysical and mechanistic studies would be required for verification, we propose that the introduced polarity at Leu377 modulates substrates affinity through changes in shape and hydrophobicity of the substrate-binding cavity, favoring product release or substrate diffusion of smaller substrates such as farnesol.

## Conclusions

In this study, we developed an automated plate-based screening platform to monitor the in vitro activity of GGR enzyme variants in a site-saturation mutagenesis library. The reduced products were recovered by a novel automated liquid–liquid extraction and acoustic printing workflow. We showed that multiple products, differing only by the degree of reduction within each isoprenyl unit, were well-separated from a homogenous mixture and resolved using thin layer chromatography (TLC). Additional efforts for workflow automation covered all aspects ranging from microbial growth to analysis, including bacterial growth optimization, expression and purification of the mutant proteins, assay initiation and quenching, and the separation and detection of derivatized products from a mixture using TLC. We successfully demonstrated that this novel TLC-based screening can distinguish fourfold differences in enzyme activity for a select L377 mutant library of SaGGR and quantitatively validated those results by GC–MS. This workflow could enable a throughput to analyze approximately ten 96-well plates per day and can be generally developed to screen a range of various reductase libraries. Also, with appropriate quantitation methods, it could enable a faster, quantitative analysis of the products at a higher throughput than traditional liquid or gas chromatography approaches.

## Methods

### Cloning and expression of SaGGR and Leu377 mutant libraries

The gene encoding SaGGR was amplified from a previously designed plasmid construct using the respective forward and reverse 5′-GATATACATATGAAGGAACTTAAATATGACGTTCTG-3′ and 5′-GTCGACGGAGCTCGAACTTAAACTTTTGTTAAACTCTGTTAGAAC-3′ primers (IDT) [21]. The gene was inserted into a kanamycin-resistant pET-24a plasmid containing a carboxy-terminal polyhistidine tag between the *Nde*I and *Sac*I restriction sites and ligated using a rapid DNA ligation kit (Roche) (Meadosw et al.). Site-saturation mutagenesis minilibraries at position Leu377 in *Sa*GGR (L377X) were generated using the forward 5′-GATTATAAAAGAAGAGGATCTG**NNK**GAAGCAAGTGAAAAAGGAGATC-3′ and reverse 5′-GATCTCCTTTTTCACTTGCTTC**MNN**CAGATCCTCTTCTTTTATAATC-3′ degenerate primers utilized for codon-saturation mutagenesis (IDT).

L377X mutants were generated by implementing the thermal cycling protocols for single-site mutations within the QuikChange II site-directed mutagenesis kit (Agilent). 1 µL of PCR products of the plasmid library was transformed into 50 µL of *E. coli* chloramphenicol-selective Rosetta(DE3)-pLysS cells (NEB) by electro-transformation. After incubation at 42 °C for 1 min followed by 2 min on ice, the transformed cells were recovered by supplementing with 900 µL of SOC medium and incubated at 37 °C and 200 rpm for 45 min. Recovered cells were subsequently plated and incubated overnight on LB-agar plates containing 50 mg/L kanamycin and 30 mg/L chloramphenicol (LB-Kan/Cm). Plasmid encoding the SaGGR WT gene (positive control) and the plasmid pET-24a (negative control) were also introduced in *E. coli* chloramphenicol-selective Rosetta(DE3)-pLysS cells (NEB) using the same method. Selected individual colonies were transferred to 96-well plates containing 100 µL LB-Kan/Cm + 10% (w/v) glycerol, incubated overnight at 37 °C and 200 rpm, snap frozen in liquid nitrogen, and stored at −80 °C for future use.

Overexpression of L377X libraries were initiated from 20 µL of overnight cultures by inoculating 1 mL of ZYP-5052 autoinduction medium containing 50 mg/L kanamycin and 30 mg/L chloramphenicol containing 0.2% v/v glucose and 0.2% (v/v) lactose. Cultures were grown in 96-deep well plates at 37 °C and 800 rpm for 24 h after inoculation, harvested by centrifugation at 3,300 RCF for 15 min, snap frozen with liquid nitrogen, and stored at -80 °C. Frozen plates containing the cell pellets were thawed at room temperature and immediately reconstituted in 500 µL of freshly prepared lysis buffer (0.1 M Tris–HCl, pH 8.0 containing 0.1 mM phenylmethanesulfonyl fluoride (PMSF) and 1 mg/mL lysozyme). The reconstituted cell pellets were lysed by incubation at 37 °C and 800 rpm for 1 h. Insoluble material was pelleted at 3,300 RCF for 15 min and the supernatant was filtered through 0.45-µm polypropylene filter plates by centrifugation at 1000 RCF for 5 min. Filtered lysate was immediately used for 96-well enzyme purification.

### 96-well SaGGR purification and activity assay

*Sa*GGR was purified from filtered cell lysate using a Beckman Coulter Biomek FX liquid handling platform equipped with modified PhyNexus Ni-IMAC tips (20 µL resin bed). The resin was stripped using 20 mM phosphate-buffered saline (PBS), pH 7.4 containing 50 mM EDTA and 0.5 M NaCl. The beads were recharged with aqueous 0.1 M CoCl_2_ to yield Co-IMAC tips. Cell lysates were bound to the Co-IMAC tips by aspirating and dispensing lysate over the resin bed for a total of five rounds. IMAC-bound SaGGR was eluted using 80 µL of elution buffer (10 mM PBS, pH 7.4 containing 25 mM EDTA and 50 mM NaCl). Protein concentration was determined immediately after purification by transferring 5 µL of protein eluent into 95 µL of Bradford reagent (Bio-Rad) and measuring absorbance against a standard curve of bovine serum albumin using a SpectraMax M2 plate reader (Molecular Devices). The remaining 75 µL of purified SaGGR were transferred to a WebSeal Plate + 96-well glass-coated microplate containing 18.5 µL of freshly prepared activity master mix (1.08 mM flavin adenine dinucleotide disodium salt hydrate (FAD) and 13.52 mM *E,E*-farnesol in 135 mM succinic acid at pH 4.0). Assays were initiated using 6.5 µL of 1 M sodium dithionite, forming a final reaction volume of 100 µL and final reactant concentrations of 200 µM FAD, 2.5 mM FOH, 25 mM succinic acid, 65 mM dithionite, and a final pH of 5.5. Reactions were covered with a WebSeal mat and placed in a 37 °C water bath for 1 h.

### SaGGR product extraction and derivatization

Liquid-phase extraction was performed using a Biomek FX liquid handling robot by transferring 95 µL of the enzyme reaction from the WebSeal Plate + 96-well glass-coated microplate into a 384-well plate pre-loaded 100 µL of a cold EtOAc:hexanes mixture (1:3, v/v). Aqueous and organic phases were homogenized by pipetting, covered with a breathable membrane, and centrifuged at 1,000 RCF for 1 min to separate phases, drawing enzyme products into the organic layer. Following centrifugation, 75 µL of the organic phase was transferred from the reaction plate into a fresh WebSeal Plate + 96-well glass-coated microplate; the solvent was freely evaporated from the microplate in a chemical fume hood.

Following solvent evaporation, the remaining unreduced isoprenoids from the enzymatic reaction were oxidized to their cognate epoxides using a modified version of previously optimized conditions [26]. The biphasic epoxidation reaction was performed by first adding 1.75 M acetonitrile (MeCN) and 25 mM 2,2,2-trifluoroacetophenone in 40 µL of *tert*-butanol (*t*-BuOH). The reaction was initiated by the addition of 6.75 M H_2_O_2_ and 40 µM EDTA in 40 µL of 0.5 M K_2_CO_3_ (pH 11) for a final reaction volume of 80 µL. Reactions were covered with a vented WebSeal mat and incubated at 50 °C and 500 rpm for 1 h.

### Epoxide extraction and application onto silica plates

Following the epoxidation reaction, 96-well plates were cooled to 0 °C on ice and centrifuged at 1,000 RCF for 1 min. Products were extracted using 100 µL of cold EtOAc:hexanes, (3:1, v/v). Aqueous and organic phases were mixed, covered with a WebSeal mat, and centrifuged at 1,000 RCF for 1 min to phase-separate epoxidation products into the organic layer. Following centrifugation, 100 µL of the organic phase was transferred from the 96-well reaction plate into 0.2 mL PCR tubes (Corning); the solvent was evaporated at room temperature. Dried products were reconstituted in 12 µL of MeCN:H_2_O (2.5:3, v/v). 10 µL was transferred to a Labcyte Echo 384-well low dead volume (LDV) microplate, referred to as the “source plate”. Source plates were centrifuged at 1,000 RCF for 5 min prior to liquid transfer onto a TLC plate, referred to as the “destination plate”. The destination plate was fabricated by affixing a 384-well LDV microplate with an aluminum-backed TLC plate having 200 µm layer thickness and a 0.79 mL/g pore volume cut to 8.0 × 11.7 cm (Merck). Prior to product application, a 10 mg/mL solution of Coomassie Brilliant Blue (Bio-Rad) in methanol:water (1:1) was printed at 1.15 cm from the bottom, and 0.70 cm from the top of the TLC plate, indicating the baseline and solvent front, respectively. These markings allowed derivatized products to migrate for a total distance of 6.15 cm. Products from the acoustic source plate were printed 1.15 cm from the TLC destination plate by acoustic droplet ejection using a Labcyte Echo 550 liquid handler at 100 nL over-spotting intervals. Following sample application, plates were dried at 50 °C for 15 min.

### Analysis by acoustic droplet ejection thin layer chromatography

For chromatographic separation, a TLC chamber with internal dimensions of 8.0 × 10.0 × 12.0 cm was loaded with 2% methanol in EtOAc:Hex (1:1.25, v/v) for a final chamber volume of 22.5 mL and allowed to equilibrate for ≥ 1 h. Single TLC plates were placed in the center of the chamber and removed once the solvent front reached the boundary defined by the Coomassie grid. The silica was dried for 30 min at 50 °C prior to development.

For plate development, resolved chromatograms were dipped in a 4% (w/v) solution of 4-(4-nitrobenzyl)pyridine (NBP) in methylene chloride (Sigma) and allowed to dry for 5 min at ambient temperature [27]. NBP alkylation was performed in situ by heating in an oven at 150 °C for 10 min. Plates were cooled to room temperature and immediately stained by dipping in 1.5% (v:v) paraffin oil in triethylamine. Product bands were imaged using a Canon CanoScan LiDE 120SC commercial scanner and library hits were identified by visual inspection.

### In vitro analysis of putative Leu377 hits

SaGGR mutants WT, L377M, L377D, L377G, L377N, L377R, and L377T were selected from the L377 site-saturation mutagenesis library to determine relative enzyme activities in vitro. From these selected clones, plasmids were extracted and SaGGR variant sequences were determined by GeneWiz (South San Francisco, CA). Selected mutants were cultured and purified using techniques developed previously [21]. Protein concentrations were determined by absorbance at 280 nm using an extinction coefficient of 81,200 M^−1^ cm^−1^. SaGGR mutant activity was performed in a 280 µL assay volume at 37 °C containing 100 mM sodium succinate, pH 5.5, 200 µM FAD, 500 µM *E,E*-farnesol, and 30 µM enzyme. Enzyme reactions were initiated with 65 mM sodium dithionite. 60 µL aliquots of the assay were removed at 0, 25, 50, and 80 min and quenched with 120 µL of EtOAc containing 100 µM dodecanol. Organic extracts were stored in GC vials containing glass inserts and stored at −20 °C until analysis.

Product identification and quantification of farnesol and its reduced products were determined by GC–MS methods previously developed using an Agilent 6890 gas chromatography setup coupled to an Agilent 5973 mass selective detector [1]. Substrate and product concentrations were quantitatively determined from a standard curve ranging from 0–200 µM. Because standards for reduced farnesol products were commercially unavailable, we assumed that the ionization intensity derived from the TIC was equal to that observed in farnesol. These intensities were converted to their equivalent number of nanomoles and plotted as a function of time to determine the specific activity of all mutants.

All structural analysis and visualization of L377X mutants were performed in Chimera (Insert UCSF reference here) using the SaGGR ternary complex crystal structure (RCSB: 4OPD) as a template [21]. Within the space filling models, the co-crystallized GGPP substrate was removed from the binding pocket to better visualize protein solvent accessibilities in the region where pyrophosphate moieties typically interact with the protein/solvent interface. All mutations were performed in silico within the Chimera platform defaulting to configurations with the lowest angular and steric strain for each mutation.

## Supplementary information

**Additional file 1.** Additional Table and Figures.

## Data Availability

Not applicable.
