# Supplementary material for: An automated workflow to screen alkene reductases using high-throughput thin layer chromatography
Source: Biotechnol Biofuels. 2020 Nov 9;13:184. doi: 10.1186/s13068-020-01821-w (PMC7653764; doi:10.1186/s13068-020-01821-w)
Supplement: Supplementary file 1 — Additional file 1. Additional Table and Figures. [file 13068_2020_1821_MOESM1_ESM.docx]

**Supplementary Information**

**An automated workflow to screen alkene reductases using high throughput thin layer chromatography**

Brett M. Garabedian^1,2,†^, Corey W. Meadows^1,2,†^, Florence Mingardon^3^, Joel M. Guenther^1,4^, Tristan de Rond^1,5^, Raya Abourjeily^3^, Taek Soon Lee^1,2,*^

^1^Joint BioEnergy Institute, 5885 Hollis Street, Emeryville, CA 94608, USA.

^2^Biological Systems & Engineering Division, Lawrence Berkeley National Laboratory, Berkeley, CA 94720, USA.

^3^Total Raffinage Chimie, 2 Pl. Jean Millier, 92400 Courbevoie, France

^4^Sandia National Laboratories, Livermore, CA, USA

^5^Department of Chemistry, University of California, Berkeley, Berkeley, CA 94720, USA

^†^These authors contributed equally to this work.

^*^Corresponding author: Dr. Taek Soon Lee, Joint BioEnergy Institute, 5885 Hollis St. 4^th^ floor, Emeryville, CA 94608, USA; Phone: +1-510-495-2470, Fax: +1-510-495-2629, E-mail: tslee@lbl.gov

**Supplementary Table S1.** Retention Factors (R_f_) for bands and their identities within the TLC-Based screen for farnesol reduction.

| Band and Identity | | Standards*^a,b^* | Rows A-D*^a,c^* | Rows E-H*^a,c^* |
| --- | --- | --- | --- | --- |
| R_f_(1) | Farnesol | 0.18 ± 0.02 | 0.18 ± 0.02 | 0.18 ± 0.02 |
| R_f_(2) | H_2_-Farnesol | 0.42 ± 0.03 | 0.38 ± 0.03 | 0.42 ± 0.03 |
| R_f_(s2) | N.D.*^d^* | 0.51 ± 0.04 | 0.45 ± 0.04 | 0.55 ± 0.04 |
| R_f_(s3) | N.D.*^d^* | 0.59 ± 0.06 | 0.51 ± 0.06 | 0.51 ± 0.06 |
| R_f_(3) | H_4_-Farnesol | 0.67 ± 0.02 | 0.63 ± 0.02 | 0.66 ± 0.02 |

*^a^*R_f_ calculated by center of each band migrates (cm) divided by migration of solvent front (cm).

*^b^*Band measurements derived from Figure 5 and Supplementary Figure 5.

*^c^*Band measurements derived from Figure 6.

*^d^*Denotes Not Determined.


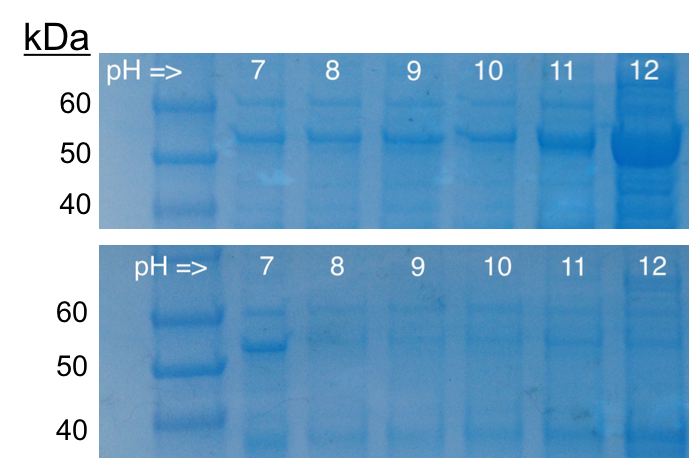


**Supplementary Figure S1.** Heat treatment of cell lysates incubated as a function of pH. As expected, increased protein recovery is observed with increased pH and buffered back to pH 7.4 (top). However, protein yields are drastically reduced after the same heat-treated lysates are buffered to the enzyme’s slightly acidic pH optimum at 5.5 (bottom).


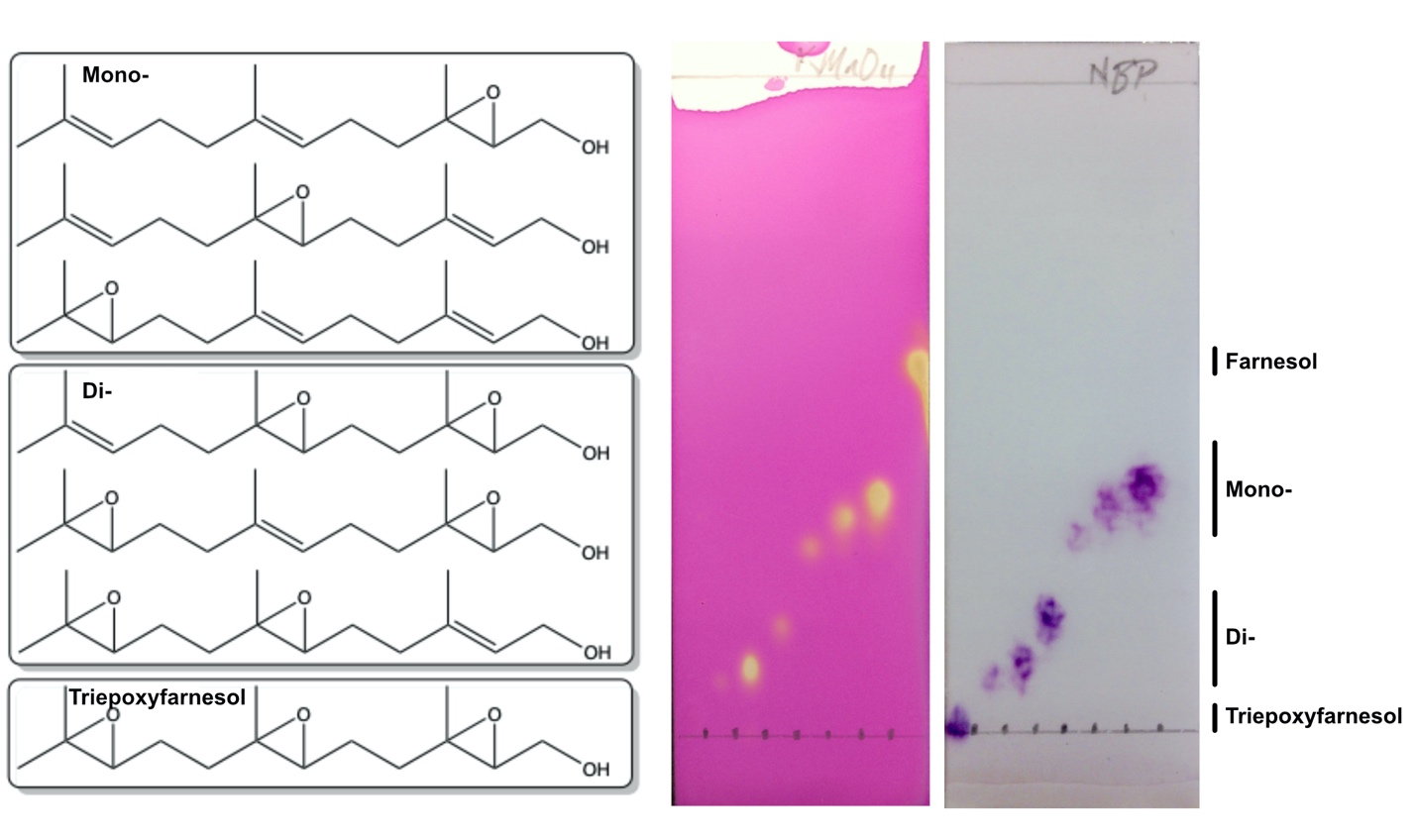


**Supplementary Figure S2.** TLC analysis of farnesol and its oxidized derivatives: mono-, di- and triepoxyfarnesol (left panel). Shown center is a plate stained using potassium permanganate to visualize prenyl groups. Shown right is an identical TLC plate stained with NBP, demonstrating the selectivity of the chromophore for epoxide products. The mobile phase was composed of EtOAc:Hexanes 1:2.


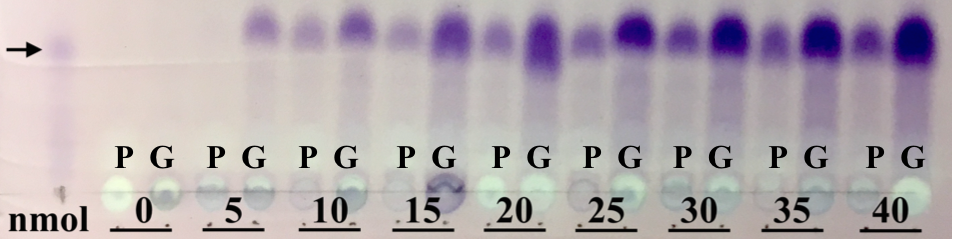


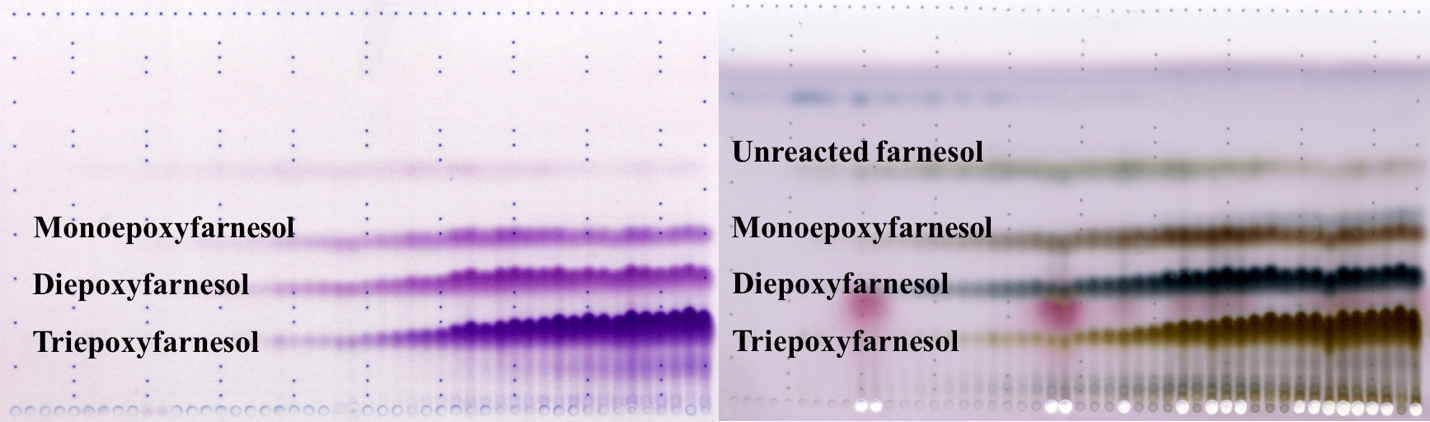


**Supplementary Figure S3.** (Top) TLC comparison of farnesol extracted after incubation for 24 hours at 50°C using polypropylene- (P) or glass-coated (G) plates as a function of initial loading amount in nmol. 1 nmol of standard FOH was ran in parallel, as shown by the arrow. (Bottom) Time course assay showing separation of partially reduced farnesol products incubated with epoxidation reagents in 96-well glass-coated plates. Products are derivatized with either NBP (left) to demonstrate epoxidation and derivatization or vanillin to reveal unreacted substrate (right). The R*_f_* for products are shown in Supplementary Table S1.

**
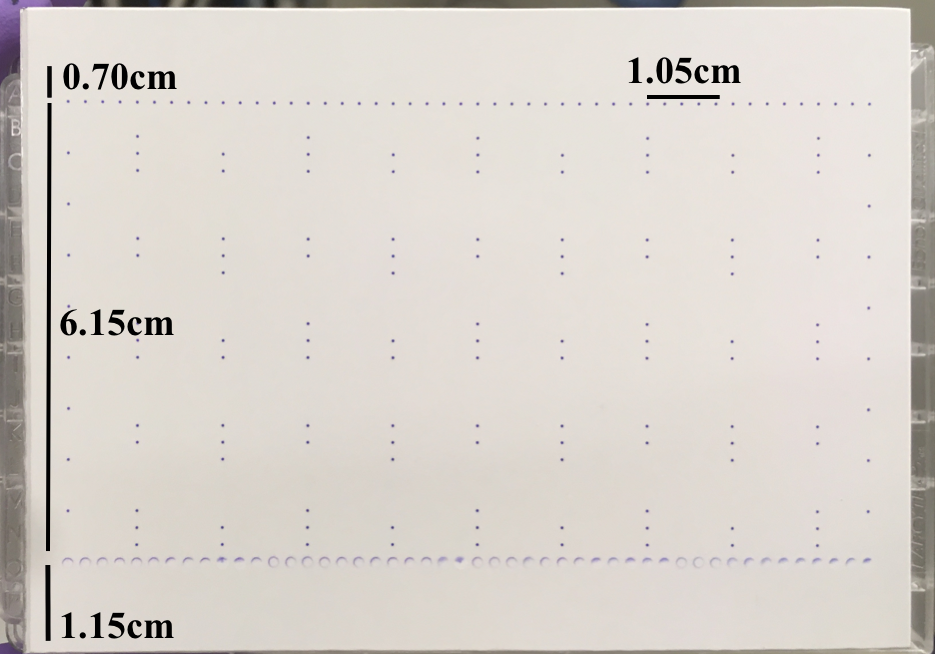
**

**Supplementary Figure S4.** Acoustic printing of epoxyfarnesol products reconstituted in 2.5:3 (MeCN:H_2_O) onto a 8.0 x 11.0 cm silica-TLC plate affixed to a 384-well low dead volume (LDV) acoustic source plate. The Coomassie stain followed product printing to be used as a guide for R*_f_* calculation, with the solvent front line printed 0.70 cm from the top and the baseline printed 1.15 cm from the bottom of the LDV plate, allowing for 6.15 cm of migratory separation. Five lanes are printed every 1.05 cm, fitting a total of 48 lanes with 0.81 cm margins. The integrity of each Coomassie spotting lane is preserved, as each circular spot’s boundaries are preserved after products are printed onto the TLC plate.


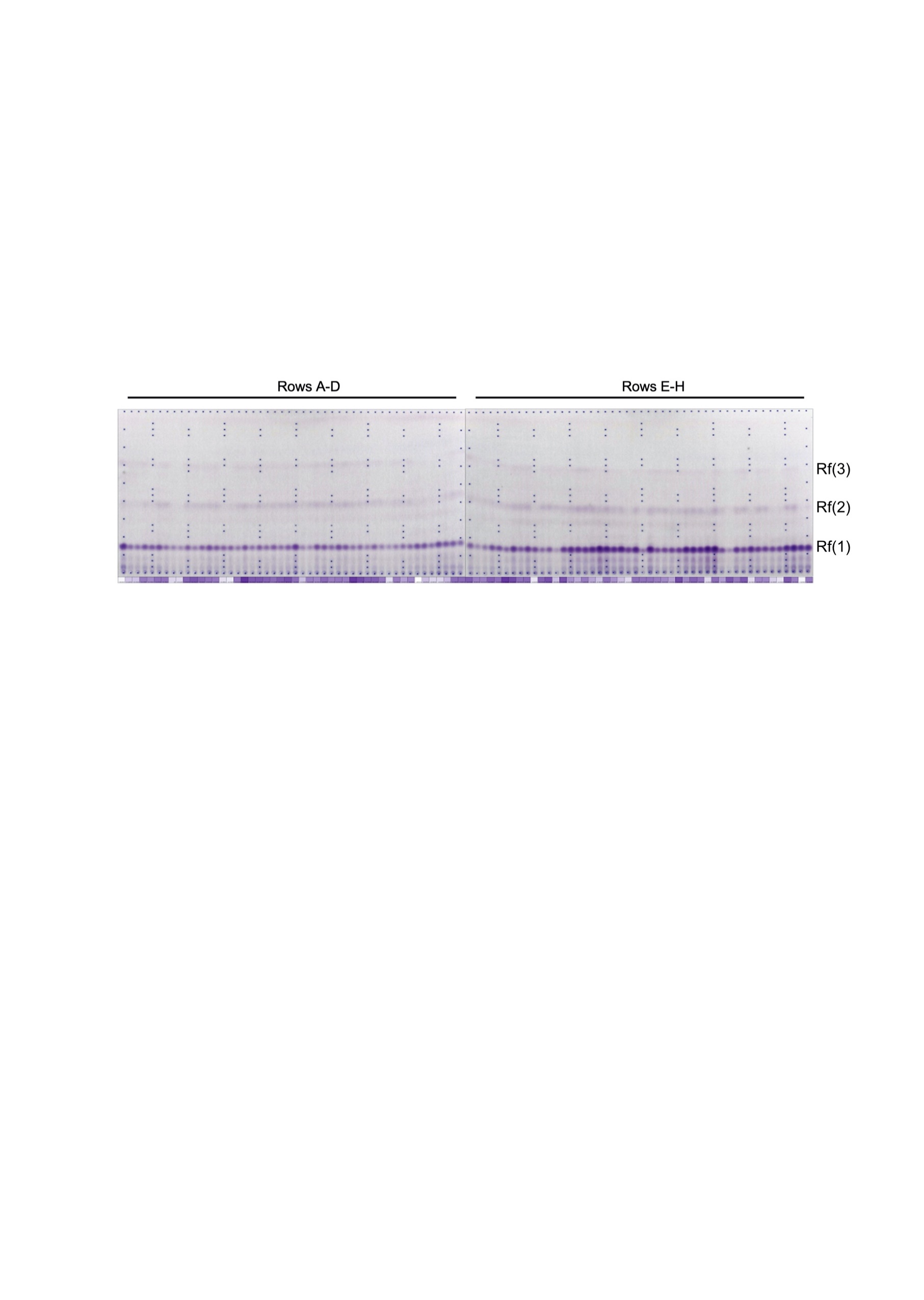


**Supplementary Figure S5.** Preliminary application of the screen to a codon saturation mutagenesis library, L377X. The first 48 lanes on the left represent activities in mutants contained in rows A-D of a 96 well plate format; the other 48 lanes on the right represent activities in mutants contained in rows E-H. Epoxide derivatives of H_0_-, H_2_- and H_4_-FOH are indicated by R_f_(1), R_f_(2) and R_f_(3), respectively. Protein concentration measured in each well via Bradford Assay is denoted under each TLC lane according to the color-coded legend in Figure 6. Following chromatographic separation, NBP-treated plates were heated in an oven at 100˚C for 10 min and developed with triethylamine.
